# Influence of site index on the relationship between forest net primary productivity and stand age

**DOI:** 10.1371/journal.pone.0177084

**Published:** 2017-05-11

**Authors:** Ying Yu, Jing M. Chen, Xiguang Yang, Wenyi Fan, Mingze Li, Liming He

**Affiliations:** 1 Northeast Forestry University, School of Forestry, XiangFang District, Harbin, Heilongjiang, China; 2 University of Toronto, Department of Geography and Program in Planning, Toronto, Ontario, Canada; 3 Northeast Forestry University, Key Laboratory of Saline-alkali Vegetation Ecology Restoration (SAVER), Ministry of Education, Alkali Soil Natural Environmental Science Center (ASNESC), XiangFang District, Harbin, Heilongjiang, China; University of the Chinese Academy of Sciences, CHINA

## Abstract

Previous studies show that forest net primary productivity (NPP) varies pronouncedly with stand age, and these variations play a crucial role in determining forest carbon sinks or sources at regional scales. Some forest carbon cycling models, eg. InTEC (The integrated terrestrial ecosystem C-budget model), calculates annual forest NPP in the long term according to normalized NPP-age relationships and the reference forest NPP at a given age. Therefore, the accurate NPP-age relationship is important for forest NPP estimation. In this study, NPP at various stand ages for twelve major forest stand types in Heilongjiang Province in northeast China is derived from yield tables with consideration of the total biomass increment and foliage and fine-root turnovers. Similar to previous studies, our results also show that forest NPP increases quickly at young ages, reaches the maximum value at middle age (10–40 years old), and then decreases to a relative stable level at old ages. However, we additionally found that forests under better site conditions have faster growth rates in young ages and steeper declines after reaching the maximum. Therefore, when the NPP-age curves for different site indices are normalized against the maximum value of each curve, there are significant differences among them. These differences have implications on the methodology for estimating the spatial distribution of forest carbon sources and sinks.

## Introduction

Net primary productivity (NPP) is defined as the difference between total photosynthesis and autotrophic respiration [1], and is an important component of the terrestrial carbon cycle. In addition to environmental factors (such as climate, CO_2_ and nitrogen deposition), disturbance (such as fire, insect and harvest) has strong impacts on the carbon cycle by directly releasing carbon to the atmosphere and by accelerating the respiratory processes [2–5]. It also alters the forest age structure and species composition, therefore modifies the spatial distribution of NPP. In general, disturbance has dominant influence on the forest carbon cycle over other factors [6–10]. To quantify the full impacts of disturbance on the forest carbon cycle, NPP-age relationships are needed for estimating regrowth after disturbance [11–15]. This is why it is imperative to analyze the controls in regulating the relationship between NPP of different forest stand types and stand age.

Different methods, e.g. forest inventory data analysis [16, 17], empirical models [6] or process-based models [18–22], have been developed to estimate forest NPP. Some models for quantifying forest NPP only consider the effects of environmental factors without taking into account of the influence of forest stand age [23–26]. However, the spatial distribution of forest carbon sources and sinks depends more on forest stand age than environmental factors [12,15, 27], and therefore it is important to consider forest stand age in carbon budget estimation [28, 29].

NPP-age curves vary among different species and are also affected by the site condition index (SCI) because forest growth depends on local soil, topographical and hydrological conditions, in addition to meteorological conditions [13, 30, 31]. SCI, defined as the average tree height at the specific age (usually at the age of 50). Younger ages are sometimes used for plantations, short-lived species, or species managed on shirt rotations [32–35]. However, so far only mean NPP-age relationships are derived under long-term mean climate conditions without consideration of site conditions. In northeast forests of China, either the constant age-related NPP curves or coarse age-NPP relationships derived using data over entire China were used for forest carbon cycle modeling [14, 21]. Fewer studies are reported on NPP-age curves under different site indices. Chen et al. [36] used stand yield tables to develop a non-linear function of age-NPP relationship stratified by a site index for several Canadian boreal black spruce forests. In northeast forest of China, age-NPP relationships are neither stratified by SCI nor developed for specific species. In this paper, we developed functions to simulate the relationships between NPP and age for twelve major forest stand types under different SCIs in northeast China, using yield tables, biomass equations for four different tree biomass components, as well as foliage and fine-root turnover rates. The maximum NPP magnitude and the age at which the maximum NPP occurs will also be analyzed against SCI. This information will be helpful for foresters to decide the most suitable curve for use in a particular area and also will be useful for carbon cycle modelers to decide specific NPP-age curves based on site condition indices.

## Methodology

### Study site and data

Heilongjiang province is located in northeast China (43°25′-53°33′ N, 121°11′-135°05′ E). It has a continental monsoon climate between temperate and boreal zones, with annual temperature of -4°C to 4°C decreasing from southeast to northwest. Its annual average precipitation ranges from 300 mm to 700 mm. Many mountain ranges, including the Greater Khinga Range, Lesser Khinga Range, Zhangguangcai Mountains, Laoye Mountains, and Wanda Mountains, dominate the land of Heilongjiang province and host the largest provincial forest resources and provide timber supply of many valuable species. These unmanaged or managed tree species such as *Pinus koraiensis*, *Pinus sylvestrisvar*, *Larix gmelinii*, *Picea koraiensis*, *Abies nephrolepis*, *Acer mono*, *Populus davidiana*, *Quercus mongolica*, *Betula davuria*, *Tilia amurensis*, and *Betula platyphylla* play an important role in carbon uptake.

Yield tables established by the Inventory and Planning Institute of Heilongjiang in 2010 based on forest inventory data from 1990–2005 were collected for stand-level NPP calculation for *Pinus koraiensis* forests, *Pinus sylvestrisvar* forests, *Larix gmelinii* forests, *Populus davidiana* forests, *Quercus mongolica* forests, *Betula davuria* forests, *Tilia amurensis* forests, *Betula platyphylla* forests, *Populus plantation*, mixed coniferous forests, mixed broadleaf forests and coniferous-broadleaf mixed forests in this study. These yield tables consist of the necessary information for estimating wood volume and biomass, and usually include age, diameter at breast height, tree height, stand density for living trees, and increment of gross total volume. The age ranges of the above stand types in yield table are 0–50, 0–50, 0–40, 0–80, 0–100, 0–70, 0–80, 0–70, 0–30, 0–150, 0–90, 0–120, respectively. Using these data, the total tree biomass (both aboveground and belowground) can be obtained by summing up its components (stem, branch, foliage, root), and used for stand NPP calculation by multiplying the tree NPP at average height and diameter with stand density. For mixed forest types consisting of various species, NPP of a forest stand is estimated based on its species composition and their areal fractions available from the inventory data, since they are not included in yield tables. The inventory data in Heilongjiang province covering the period of 1990–2005 involve 19,500 permanent field samples that provide growth, site information, species composition, stand age, forest type, stand density, site condition index and so on. Site condition index is calculated according to national standard, “the main forest types harvest table in cities and countries” (Number: DB23/T 1377–2010), in China. It is quantified by Eq 1 for *Pinus koraiensis* and *Pinus sylvestrisvar* forests, by Eq 2 for other stand types. Here, *SCI* refers to site condition index, *t*_*1*_ refers to stand specific age, k and c are parameters. Stand specific age refers to the age at where stand growth trends to be stable. Average stand height is calculated by Eqs 3 and 4 (Eq 3 for *Pinus koraiensis* forests and *Pinus sylvestris* forests, Eq 4 for other stand types, where *TH* refers to average stand height, *t* refers to stand specific age, A, k and c are parameters). Parameters used in the above model were obtained from yield table compilation calculating from inventory data (Table 1). The larger value of site condition index indicates better site condition.

$$SCI=TH\text{ exp}\left[-k\left(1/t_{I}-1/t\right)\right]$$ (1)

$$SCI=TH\frac{{\left[1-\text{exp}(-kt_{I})\right]}^{c}}{{\left[1-\text{exp}(-kt)\right]}^{c}}$$ (2)

TH=A exp(−k/t) (3)

$$TH=A{\left[1-\text{exp}\left(-kt\right)\right]}^{c}$$ (4)

**Table 1:** Parameters used in Eqs 1 to 4.

Stand types	A	k	c	t_1_
*Pinus sylvestrisvar forests*	19.253	17.45400	-	30
*Mixed coniferous forests*	42.148	0.003672	0.624529	80
*Coniferous-broadleaf mixed forests*	26.534	0.007491	0.579309	50
*Quercus mongolica forests*	19.061	0.011785	0.579212	50
*Populus plantation*	16.686	0.041108	0.620218	20
*Populus davidiana forests*	21.006	0.032326	0.805220	30
*Mixed broad-leaf forests*	23.833	0.007678	0.537825	50
*Larix gmelinii forests*	24.725	0.023100	0.836503	30
*Pinus koraiensis forests*	18.330	21.45826	-	30
*Betula davuria forests*	18.745	0.020605	0.775345	40
*Tilia amurensis forests*	17.485	0.028606	0.837695	50

### NPP calculation

In this study, NPP is calculated based on normal yield tables, biomass allometric equations, inventory data, and leaf and fine-root turnover rates from other studies. NPP is modeled as the sum of biomass increments, litter-fall and fine-root turnovers [36] for twelve forest types in Heilongjiang province, northeast China:

$$NPP=\frac{dB}{dt}+L_{f}+L_{fr}$$ (5)

where *dB/dt* is the increment of dry matter in total living biomass per year at the specific age with the assumption that the fine root don’t change in two successive years, and it is estimated from the yield tables; *L*_*f*_ and *L*_*fr*_ are the annual foliage and fine-root turnovers. The methods to compute the biomass increment in living trees and foliage or fine-root turnovers will be described in the following paragraphs. Understory NPP and tree mortality are not included in this NPP estimation because of the lack of understory vegetation data and mortality statistics.

In this study, the total biomass (*B*_*t*_) is separated into four components: *B*_*s*_ for stem, *B*_*b*_ for branch, *B*_*f*_ for foliage, and *B*_*r*_ for coarse root. Dong et al. [37] developed empirical models for the total biomass and the biomass of six components including aboveground, underground, stem, crown, branch, and foliage biomass for 15 major tree species (or species groups) based on a non-linear error-in-variable modeling approach and 516 sampling trees in Heilongjiang Province. These models are adopted in our study. Models and their coefficients for eight species are provided in appendix A and Table 2. The accuracy rate for biomass estimation was not calculated in this work, it was from the results of Dong’s work [37], since the estimation of biomass was used by the models in his work. In order to avoid the infinite increases of foliage biomass estimation from some of the empirical functions with limited sample ranges of stand age, we made an assumption that the maximum foliage biomass appears at the stage of fastest growth of living biomass.

**Table 2:** Compatible model and its corresponding coefficients.

Species	Total biomass *B*_*t*_ (Eq 10)			Aboveground biomass *B*_*a*_ (Eq 11) Root biomass *B*_*r*_ (Eq 12)			Stem biomass *B*_*s*_ (Eq 13) Crown biomass *B*_*c*_ (Eq 14)			Branch biomass *B*_*b*_ (Eq 15) Foliage biomass *B*_*f*_ (Eq 16)	
	lna_1_	b_1_	c_1_	r_1_	r_2_	r_3_	r_1_	r_2_	r_3_	r_1_	r_2_
*Pinus sylvestrisvar*	-3.12	3.23	0.51	0.34	-0.49	0.35	0.18	1.22	-1.30	2.39	-0.40
*Larix gmelinii*	-2.22	2.00	0.51	0.31	0.79	-0.86	5.6	0.09	-1.32	2.78	-0.77
*Pinus koraiensis*	-2.41	2.47	-	0.43	0.63	-0.94	0.13	1.10	-0.85	7.32	-0.86
*Betula davuria*	-2.73	2.29	0.37	1.47	0.04	-0.74	0.06	1.88	-1.53	0.68	-0.35
*Tilia amurensis*	-3.17	1.88	0.91	1.88	-0.90	0.26	0.52	0.41	-0.86	5.60	-1.08
*Betula platyphylla*	-2.89	2.08	0.69	1.05	0.48	-0.94	0.31	0.94	-1.01	1.24	-0.61
*Populus davidiana*	-4.09	2.36	0.72	3.74	0.11	-1.14	0.01	1.59	-0.66	10.54	-1.26
*Quercus mongolica*	-2.92	2.21	0.63	1.62	0.51	-1.27	0.11	1.41	-1.22	1.70	-0.75

Note: D is the average diameter at breast height; H is the average tree height

This method solves the problem that the sum of four components of biomass does not equal to total biomass. In these models, the tree diameter at breast height and the tree height are taken as independent variables affecting each biomass component. We use these models to calculate total tree biomass in chronological series in yield tables for each species. Stand biomass per unit area varies with age and is estimated by multiplying the single tree biomass with tree density.

**Fig 1:**
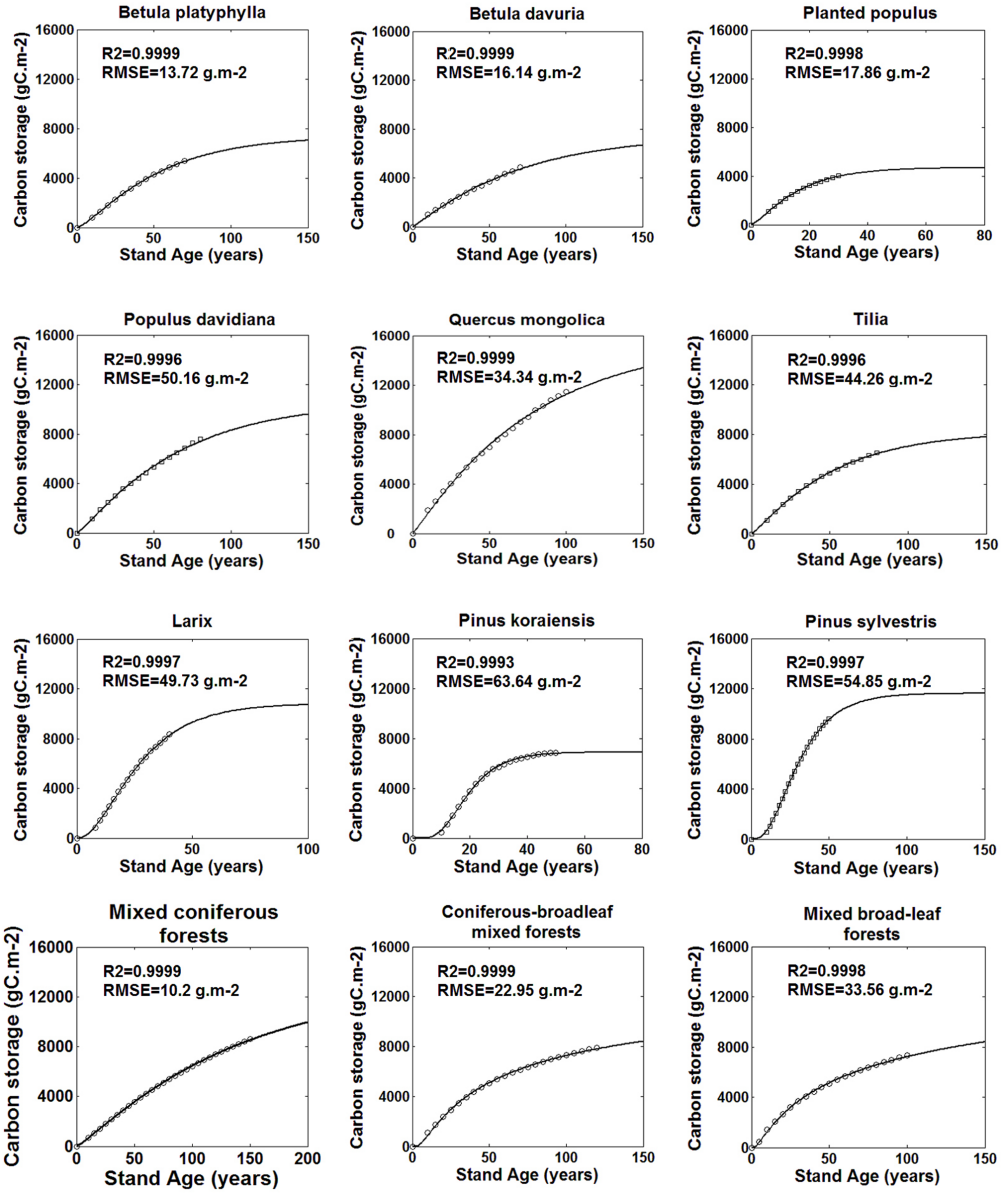
Total biomass changes with age.

Plant growth equations are selected to model the biomass changes with age. Many different growth functions were established in previous studies, including Logistic equation, Mitscherlich equation, Gonmpertz equation, Korf equation and Richard's equation [38–43]. All these equations show a growth pattern like “S” curve, i.e. the slow-fast-slow-stable pattern. Here, 5 growth models were used separately to fit the relationship between biomass and age for different species in Heilongjiang Province. It was not convergence for parameter estimation by using Mitscherlich equation for almost all species except for *Pinus koraiensis*. Korf model cannot be used to fit the biomass-age relations of *Pinus koraiensisvar* since it also has convergence problems. However, for coniferous-broadleaf mixed forest, Korf model was the best with the minimum root mean square error (RMSE) and maximum determination coefficient (R^2^) of 38.76 g•m^-2^ and 0.9998. The fitting precisions of the Logistic, Gonmpertz and Richards models did not appear much difference. In order to avoid the effects of uncertainty of biomass estimation by using different models on NPP results. Richards equation (Eq 6) with the average minimum RMSE and maximum R^2^ was chose at last. RMSE and R^2^ of Richards model for all species were showed in Fig 1.

$$B=A{\left(1-exp(-rt)\right)}^{c}$$ (6)

where *B* is the estimated biomass (g•m^-2^) as a function of stand age (*t* in years) and its true value is calculated from *B*_*t*_. *A*, *r*, and *c* are parameters representing the maximum biomass, growth rate and assimilating capacity and can be determined as coefficients of biomass-age models (Eq 6). Furthermore, various nutrition conditions, climate changes and management policies impact tree growth curves. However, the average diameter and height inputs to the growth equation represent the smooth growth under average environmental conditions. In previous studies, the average annual biomass increment was taken into account for the first part of NPP calculation in Eq 5 that may smooth the growth rate of plants or even miss the important change at certain stages, and was not a successive increment changing with age. Therefore, *dB/dt*, the derivative of the growth equation, is better to capture small changes in the plant growth rate.

Litter-fall and fine-root turnovers are the other two components of NPP and are estimated separately because they are not parts of the yearly biomass increment. Litterfall is quantified as age-dependent foliage biomass times its corresponding turnover rate (Eq 7). For deciduous broad-leafed species, obviously their foliage turnover rates are equal to 1. However, for coniferous species, the needle life span varies in relation to many factors, including temperature, light, water, nutrients, insects, diseases, and air pollution [44], and is rather difficult to measure or quantify. We use specie-specific leaf turnover rates published in White et al. [45]. Fine-root turnovers are estimated according to the foliage turnovers by introducing the allocation ratio between new fine root C and new leaf C (Eq 8). The principle for linking leaf and fine-root turnovers is presented in Thornton (1998). Values of the ratio of new fine root carbon to new leaf carbon allocation and foliage turnover rates are chosen from White et al. [45] for different species (Table 3).

$$L_{f}=B_{f}\times T_{f}\times C_{f}$$ (7)

$$L_{fr}=L_{f}\times e$$ (8)

Where *T*_*f*_ is the foliage turnover rate (yr^-1^); *C*_*f*_ is the ratio of carbon to dry matter; and *e* is the allocation ratio between new fine-root carbon and new leaf carbon. We use 0.44 for *C*_*f*_ according to our previous work on forest carbon ratios among biomass components in Northeast China forest area [46]. The carbon content coefficient of the total tree was 0.45.

**Table 3:** Values of new fine root carbon to new leaf carbon allocation and foliage turnover rates for different species.

Species	Leaf turnover rate *T*_*f*_ (yr^-1^)	New fine-root carbon to new foliage carbon ratio *e*
*Pinus sylvestrisvar*	0.385	1.03
*Larix gmelinii*	1	1.4
*Pinus koraiensis*	0.4^a^	1.4
*Betula davuria*	1	1.26
*Tilia amurensis*	1	1.2
*Betula platyphylla*	1	1.26
*Populus davidiana*	1	1.2
*Quercus mongolica*	1	1.2

^a^leaf turnover rates used for *Pinus koraiensis* was published in Liu et al. (5)

The relationship between NPP calculated from the above methods and forest stand age from yield tables is modeled using Eq 9, where *M*, *b*, *d*, and *g* are coefficients to be determined [12]. Normalization of a NPP-age curve against the maximum NPP produces the pattern of NPP variation with age and can be used in carbon cycle models to reconstruct the historical NPP variation with age.

$$NPP(age)=M[1+\frac{b{\left(age/g\right)}^{d}-1}{exp(age/g)}]$$ (9)

## Results and discussion

### Age effects on biomass and NPP

**Fig 2:**
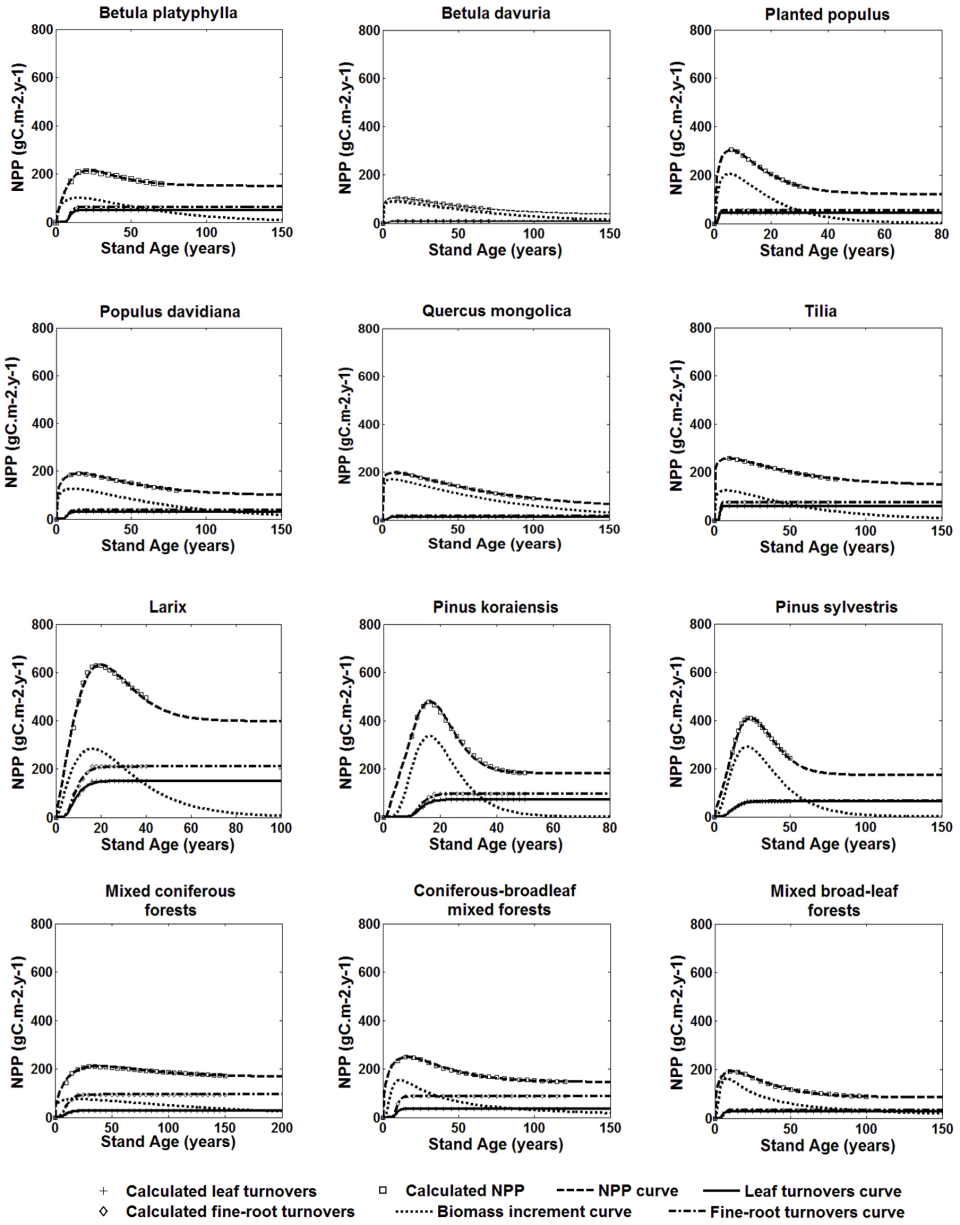
NPP and its components change with age.

The variations of the total biomass, total NPP and its components with stand age under the average site condition index (SCI = 14) for each stand types in Heilongjiang Province are shown in Figs 1 and 2. The curve of the total biomass changing with age is well fitted using Richard’s equation. NPP increases rapidly before reaching its maximum and thereafter decreases to a relatively steady state. At younger ages, carbon is mostly accumulated in stems, branches and coarse roots so the total NPP is dominated by living biomass increments. The decline of NPP with age is mainly caused by the decreasing rate of living biomass increment. At older ages, NPP-age curves are dominated by leaf and fine-root turnovers since carbon allocations to these two components are larger than the other parts. The stable state of NPP is due to the assumption that leaf turnovers remain constant after reaching the maximum value. Our previous study shows that foliage turnovers decline a little at older ages for many stands [13]. Therefore, we realized that this assumption should be examined further when chronosequences of litter-fall data are available.

It is shown from Fig 2 that different stands have distinct NPP magnitudes and NPP-age patterns. The maximum NPP showed large variations among different stands, ranging from 108 to 631gCm^-2^yr^-1^. For pure broad-leaf forest, the maximum NPP ranged from 108 to 260 gCm^-2^yr^-1^, with the lowest value for *Betula davurla*stands (108 gCm^-2^yr^-1^). While for pure coniferous forests, the value changed between 303 to 631 gCm^-2^yr^-1^ with the highest value for *Larix gmelinii* stands (631gCm^-2^yr^-1^). The maximum NPP of coniferous forests is much larger than that of broad-leaf forests in pure forest stands, while plantations are more capable to sequester carbon than natural forests. The carbon accumulation capacity of coniferous-broadleaf mixed forests is greater than those of mixed coniferous and mixed broad-leaf forests, with the maximum value of 256 gC m^-2^ yr^-1^. Coniferous forests grow to mature more slowly and its NPP reaches the maximum value at later ages, i. e. 20 years or more. NPP of *Pinus sylvestrisvar* stand reaches the maximum at 30 years. NPP of broad-leaf forests reaches the maximum at younger ages, i. e. 10 year old. Among three natural mixed forests, the mixed broad-leaf forests reach the maximum NPP earliest at the age of 17 years, followed by 23 years for coniferous-broadleaf mixed forests and 43 years for mixed coniferous forests. The carbon sequestration in coniferous-broadleaf mixed plantations can be two times or more greater than natural coniferous-broadleaf mixed forests, similarly to results of Thomas et al. [47]. Wang et al. [14] showed that forest NPP reaches its maximum at an older age than that of our study, possibly because, (1) they studied five forest biomes over the China, which are incomparable to the few specific species within Heilongjiang province with only 37 samples of all species, and (2) the forests in Heilongjiang grow to mature earlier with fertile soil.

### Effects of age and site index on volume increment

**Fig 3:**
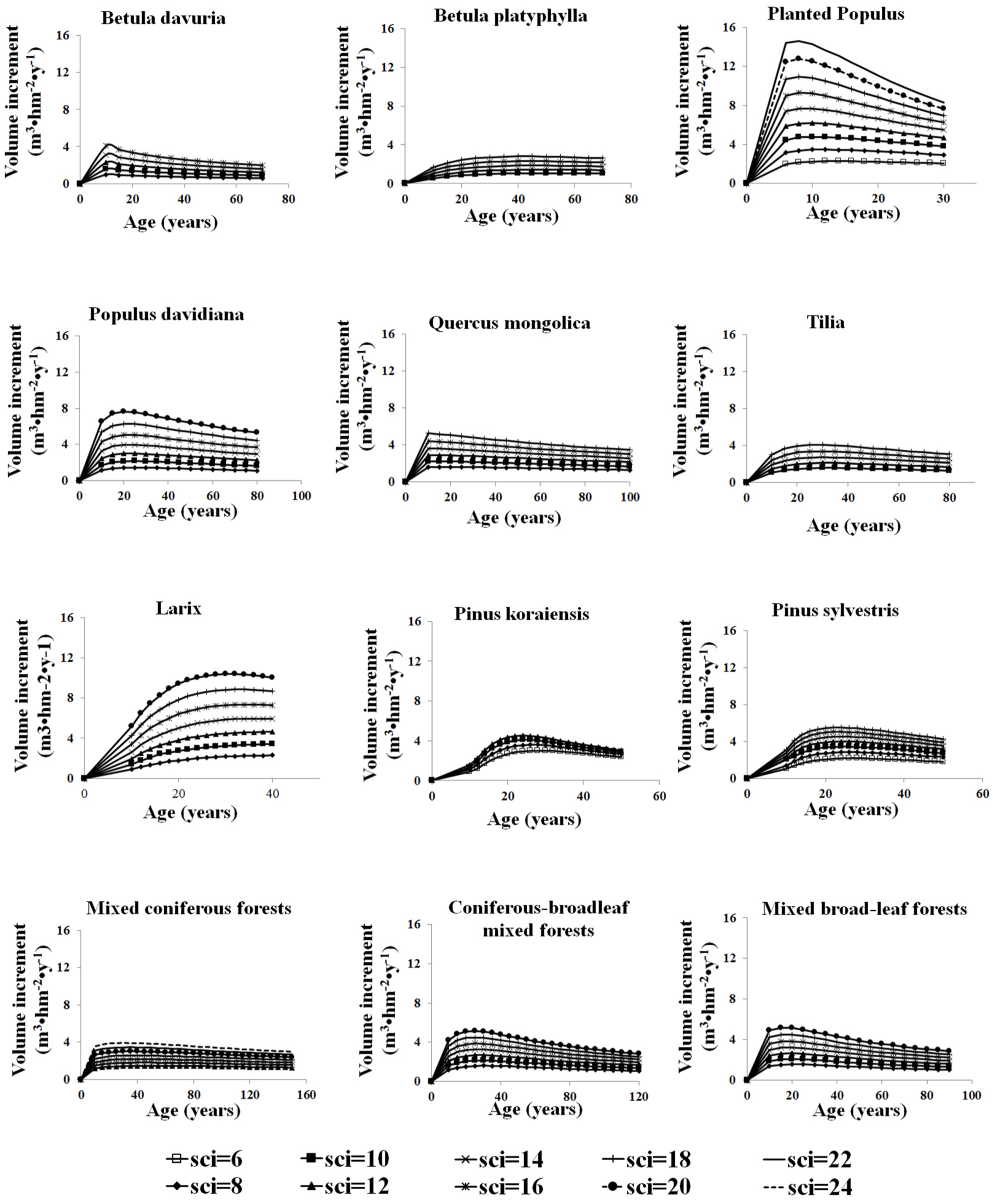
Relationship between volume increment and age under different site condition index.

The volume increment is defined as the average volume growth of a stand within a certain time period and it varies with stand age. Fig 3 shows age-related volume increments derived for twelve stand types under various SCIs. Volume increments at different ages are estimated from the yield tables for stands of 10–150 years old. For all species, the better site condition, the larger is the volume increment and the greater are the increment changes around the peak age. Based on the classification of sites and their corresponding volume increment curves, we can infer their capacities to produce wood. These volume increment curves for each species are not proportional to the site condition index, suggesting that we should stratify proper volume increment data according to the site condition index before analyzing the data. In conclusion, both the SCI and age affect the volume increment and are important to carbon cycle estimation.

Volume increment is a useful indicator for biomass increment since many studies show that timber volume is linearly related to biomass. It is also a measure of growth status influenced by environmental factors, such as water content, nutrient condition, radiation and temperature. Among different stand types, there is a large difference in the volume increment. *Populus davidiana*, the fastest volume growth among all broad-leaf natural forests, reaches the maximum volume increment earlier than *Betula platyphylla* and *Tilia amurensis*. *Populus* plantation is two times more productive and two times faster than natural *Populus davidiana* to reach the peak. This is perhaps due to forest management. *Larix gmelinii* plantations have more volume increments and reach the maximum values later than *Pinus koraiensis* and *Pinus sylvestris var*. For natural mixed forests, the maximum volume growth of mixed coniferous forests is smaller than coniferous-broadleaf mixed and mixed broad-leaf forests. Three types of mixed forests have the largest volume growths at different ages: about 17 years old for mixed broad-leaf stands and 20–30 years old for mixed coniferous and coniferous-broadleaf mixed stands. However, the relationships between volume increment and age can only explain the volume or woody biomass growth, or show the discrepancies in the carbon uptake abilities among trees of the same mono species under different environmental conditions. A large amount of volume growth for some stands does not always indicate more carbon accumulations. Additionally, NPP may be large in small volume growth stands, or small in large volume growth stands because it is affected by many factors in addition to volume growth, such as carbon to dry matter ratio, and leaf and fine-root turnover rates. Therefore, it is necessary to calculate NPP for carbon uptake estimation and comparison among different stands under different SCIs.

### Effects of site index on NPP-age and normalized NPP-age curves

**Fig 4:**
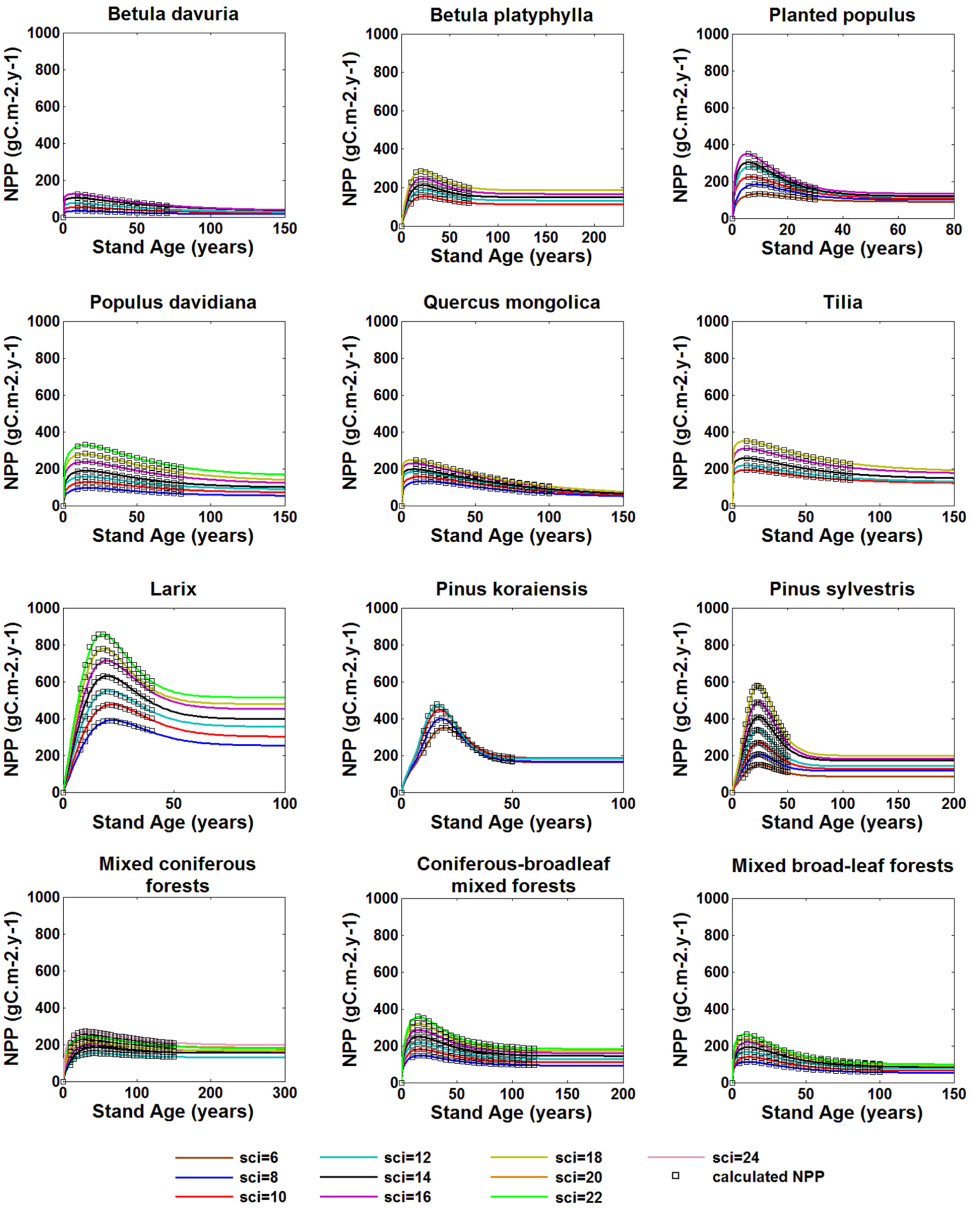
Relationships between NPP and age for different species under various site condition indices.

Stand NPP changes with age under different SCIs as shown in Fig 4 and have similar patterns to relationships between volume increment and age. Better site conditions produced greater NPP, faster growth rates in young forests and steeper declines after reaching the maximum value. The peak age is smaller in better site conditions. This general pattern is consistent with results of Chen et al. [36].

The site condition index has a great impact on mono-species stand NPP. The changes of NPP with age are not proportional to SCI. The largest NPP variations caused by SCI differ from 50% to 500% indicating that more attention should be paid to different site conditions index for determining NPP-age relationships. The relative variations of NPP with age at poor sites are smaller than those at good sites. Therefore, it is necessary to choose a suitable NPP-age curve for carbon cycle modeling based on SCI in order to get more accurate estimation of NPP if the spatial distribution of SCI is available in the future for large areas, and it is helpful for the foresters to use SCI to decide the most suitable NPP-age curves for forest management in a particular area.

**Fig 5:**
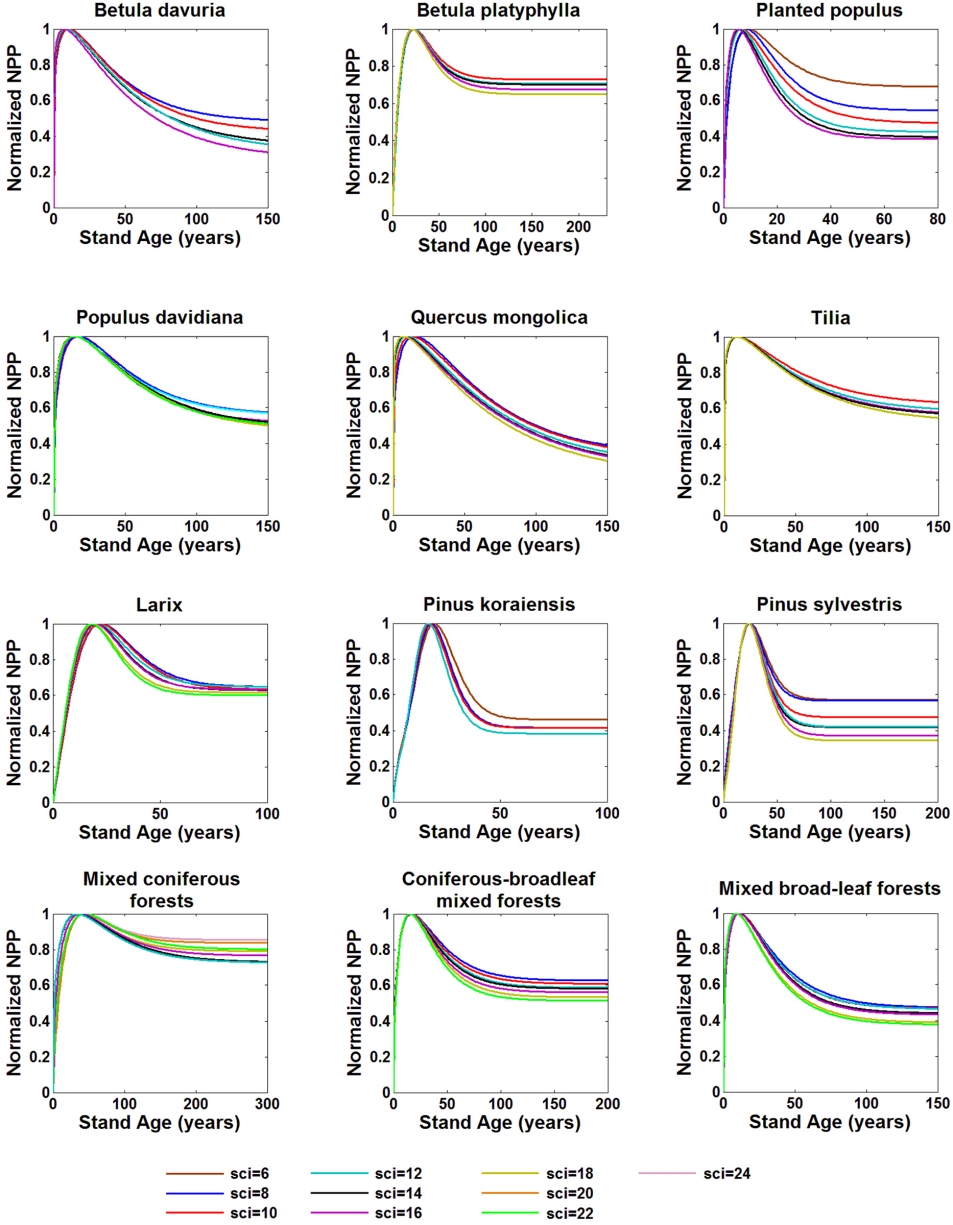
Normalized NPP-age curves for different species under various site condition indices.

In regional carbon cycle estimation, the absolute NPP value in a pixel in a recent year can be modeled based on remotely sensed LAI and land cover information in combination with soil and meteorological conditions [48], while normalized NPP-age relationships are used to extend this NPP value to other years in long-term carbon cycle modeling [12]. The normalization is made by dividing a NPP-age curve by the maximum NPP in the curve. It is therefore of interest to know if the NPP-age curves under different site condition indices can be normalized to one curve. Normalized NPP-age relationships under different SCIs for twelve kinds of stands are shown in Fig 5. Comparing to the absolute NPP-age relationships (Fig 4), the normalized ones are much less sensitive to SCIs. The normalized NPP-age variations under different SCIs are within 10% for most stands except for *Populus* plantation and *Pinus sylvestrisvar* forests, which differ by about 30% and 22%, respectively, across different SCIs at old ages. Interestingly, the same variation is not noticed for natural *Populu*s *davidiana* forests. Since NPP values of the same mono specie forests at old ages, whether planted or natural forests, are more influenced by physiological limitations than forest management, the planted and natural *Populus davidiana* have the similar NPP values at old ages. Meanwhile, *Populus* plantations grow faster than natural *Populus davidiana* forests, and produce much more NPP at growing boom ages under the same site condition. Therefore, the differences in normalized NPP curves of *Populus* plantations are larger than that of natural *Populus davidiana*. Unlike the *Populus davidiana*, *Pinus sylvestrisvar* forests have longer life span, and are more adaptable to site conditions, more resistant to cold and drought conditions. Under poor site conditions, NPP of *Pinus sylvestrisvar* at old ages does not change too much from that under good site conditions (Fig 4), whereas at peak growing stages, NPP is much larger under better site conditions. Thus, *Pinus sylvestrisvar* has more different normalized NPP-age curves under different SCIs than those of other coniferous species. Comparing to mono specie forests, the variations of NPP-age relationships with SCIs of coniferous-broadleaf mixed forests was the smallest. For the three types of mixed forest stands, the normalized NPP-age curves vary less than 10% under different SCIs. These results suggest that the NPP-age curve normalization is an effective way to extract the general NPP-age curve shape for a species or a forest type for regional carbon cycle modeling. Applying only one normalized curve for a stand type to different SCIs could generally incur an error in NPP estimation at old ages by less than 10%, but for some stands, the error can be as large as 30%. More studies are needed to quantify this error.

**Fig 6:**
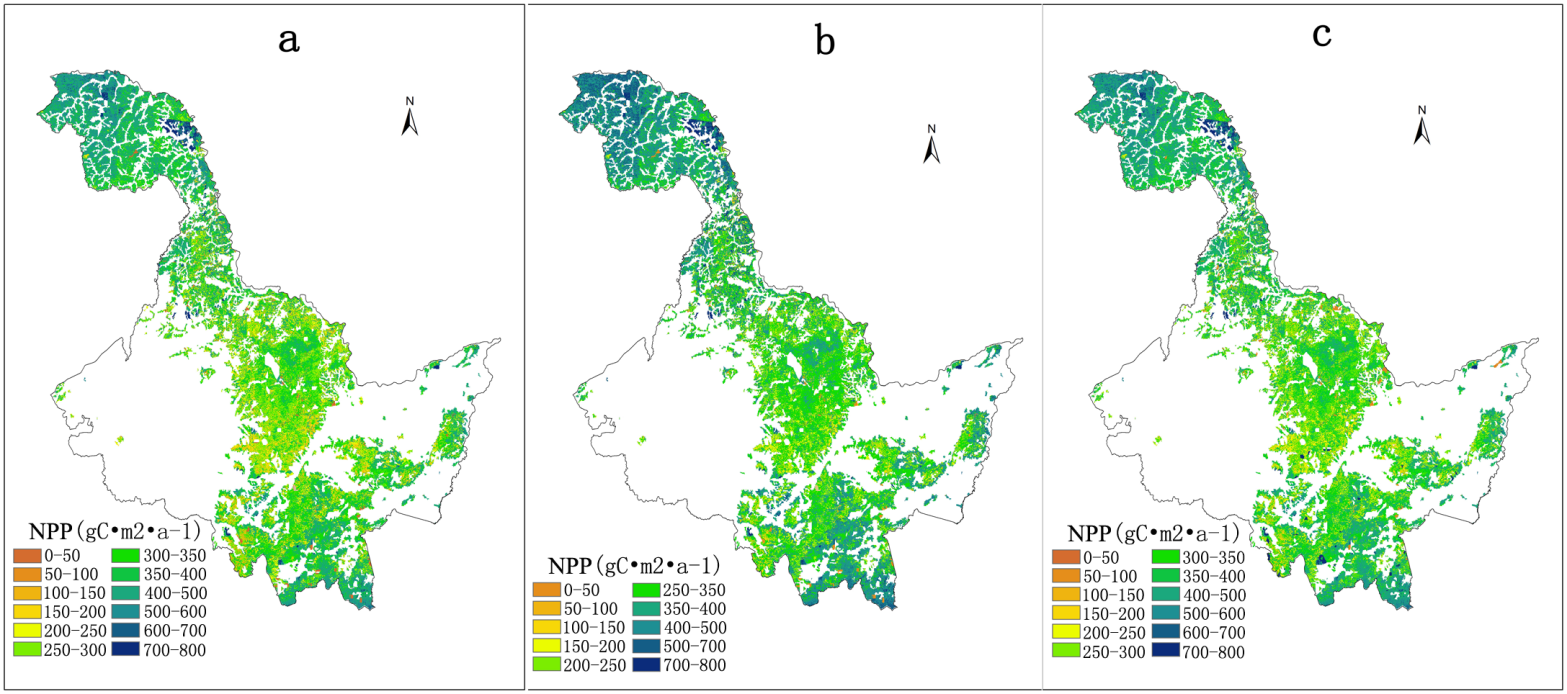
NPP estimation based on NPP-age relationships at different site condition indices, SCI = 12 (a), SCI = 14 (b), SCI = 16 (c).

For most carbon cycle models, historical NPP was calculated based on constant NPP-age relationship, ignoring the effects of site conditions. In this study, we choose NPP-age relationships under different site condition indices (SCI equals to 12, 14 and 16) to estimate NPP in 2009 (Fig 6(a), 6(b) and 6(c)) and analyze the differences. Some NPP changes can be found in different SCIs shown in Fig 6. Forest NPP at old ages in southern and northern parts of Heilongjiang province shows a slight reduction as SCI increases. However, in the middle of Heilongjiang province, forest NPP at younger ages shows some increases when site condition becomes better. These results also suggest that NPP under better site conditions has faster increases at younger ages and rapid decreases at old ages, and has larger relative changes than NPP under poorer site conditions.

### Relationships between NPP and age for different stands

**Fig 7:**
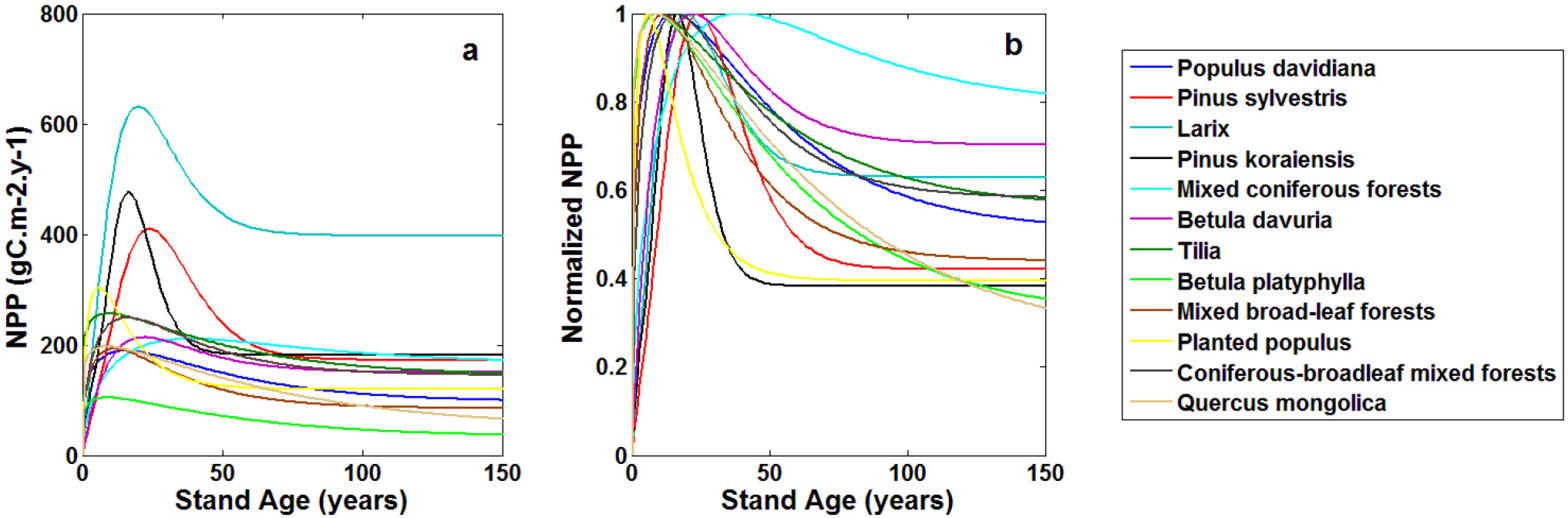
Relationships between NPP and age (a), normalized NPP and age (b) for different species under mean site condition index (SCI = 14).

NPP-age curves under the mean SCI (SCI = 14) are shown in Fig 7(b). The fitted coefficients for Eq 9 and R^2^ (determination coefficient) for specie-specific functions are listed in Table 4. Pure coniferous plantations have larger NPP than natural broad-leaf forests. Natural coniferous forests can accumulate more carbon than natural mixed broad-leaf and mixed coniferous forests. Coniferous forest NPP decreases substantially after reaching to its maximum value, while the decrease is not as pronounced for broad-leaf forests except the *Populus* plantations. These results are similar to those of He et al., [13]. NPP of *Populus* plantations shows an obvious decline in old ages. More productive species have greater changes of NPP with age and show a quicker increase to the maximum and steeper decrease afterwards. Our study shows that NPP of old forests (> 100 years) maintains about 30–60% of the maximum NPP occurred at mid-ages (15–40 years). Previous studies indicated that NPP in old forests generally decreases to about half or one-third of its maximum value [49]. Gower et al. [50] stated that the aboveground NPP commonly decreases by 0–76% as stands mature, similar to the result of about 0–63% decreases by He et al., [13].

**Table 4:** Coefficients of age-related NPP functions for different stands for Eq 9.

Stands	M	b	g	d	R^2^	RMSE	precision
*Pinus sylvestrisvar*	172.477	0.628	6.852	3.437	0.9961	5.326	95.77%
*Mixed coniferous forest*	166.656	1.712	35.041	0.495	0.9938	6.322	95.24%
*Coniferous-broadleaf mixed forest*	144.989	2.888	20.944	0.455	0.9980	3.655	98.45%
*Quercus mongolica*	48.000	5.808	55.391	0.122	0.9998	2.176	99.17%
*Populus plantation*	120.076	4.789	9.420	0.458	0.9998	2.753	98.90%
*Populus davidiana*	96.369	3.176	32.026	0.292	0.9997	3.012	98.75%
*Mixed broad-leaf forest*	84.991	4.048	20.142	0.388	0.9975	4.760	96.77%
*Larix gmelinii*	397.404	0.803	7.186	2.513	0.9969	5.094	95.80%
*Pinus koraiensis*	181.997	0.053	3.147	5.228	0.9982	3.579	98.63%
*Betula davuria*	32.278	4.720	43.153	0.147	0.9999	2.086	99.73%
*Tilia amurensis*	143.107	2.394	38.398	0.122	0.9997	2.997	98.83%
*Betula platyphylla*	150.000	1.892	14.113	1.050	0.9978	4.962	96.85%

The decrease of NPP at old ages is mainly due to the declining carbon allocation to wood components, in addition to increased autotrophic respiration for sapwood maintenance, decreased photosynthesis efficiency and declining N-availability to trees [49]. In addition to these factors affecting the performance of individual trees, changes in forest structure, such as self-thinning and wind damage, would also negatively impact forest NPP at old ages [51]. For old age forests, leaf and fine root turnovers take a large part of photosynthetic productions [52]. Accurate estimates of leaf and fine root turnovers and carbon allocation ratio of new fine roots to new leaves are of importance to NPP calculation.

Generally, the fine root turnover consumes a larger proportion of net primary productivity in coniferous forests than that in broadleaf forests. Our study shows that it costs 5.6%~36.3% and 5.8%~37.8% of NPP for leaf and fine root turnovers in *Pinus sylvestrisvar* plantations at age older than 10. These values for *Pinus koraiensis* plantations, broadleaf forests are 2%~40.3% and 2.7%~56.4%, 13.7%~31.3% and 16.8%~38.2% respectively. These values not only vary with site condition indices but also with forest ages. *Larix gmelinii* plantations allocate 32.5% of NPP to fine roots at 20 years old under the mean site condition index (SCI = 14). It’s larger than that in Mei’s work, that fine root consumed about 12 percent of NPP in *Larix gmelinii* plantations [53]. This is possibly because: (1) the calculation of fine roots in her work only includes fine roots with the diameter less than 1 mm, while the definition of the fine root diameter in our study is less than 2 mm; and (2) the site condition chosen in Mei’s study is better than that of the mean SCI. Larger fine root turnovers occur under worse site conditions since it can produce more nutrients. Other research showed that fine root turnovers returned 18.1% of NPP to the soil carbon in *Larix gmelinii* plantations, while litterfall returned 18.4%to the soil carbon, nearly the same [54]. It means the carbon allocation ratio between new fine root and new leaf equals 1, smaller than the value 1.2 used in our study. So far, there are many studies on the estimation of forest fine root productions and turnover rates. However, few are about the carbon allocation ratio between new fine root and new leaf, especially for northeast forests of China. Therefore, further field measurements focused on this issue would be beneficial to forest carbon cycle research.

The normalized NPP-age curves under mean site condition index (SCI = 14) as shown in Fig 7(b) are useful for regional carbon cycle modeling. Some process-based models, such as the Integrated Terrestrial Ecosystem Carbon (InTEC) model [55], use normalized NPP-age relationships but not the absolute NPP values. These normalized curves accentuate the NPP-age variation patterns of different species, and a curve can be used to reconstruct historical stand dynamics based on NPP at a time with a known stand age [12, 27]. From the perspective of ecosystem carbon uptake, the capacity of plantations is higher than natural forests since older forests in protected natural areas tend to reach carbon neutrality at older ages. However, pure plantations may encounter the problems of pest, fire and wind disturbances easily. It is therefore more advisable to consider planting coniferous-broadleaf mixed forests in northeast area of China.

## Uncertainty analysis

The model for total living biomass has the highest accuracy at about 90%, followed by the model for stem biomass with an accuracy of 87.5% [37]. The accuracies of the biomass models for coarse roots and foliage are relatively low, but are still greater than 80% for all species. The method to obtain the sum of three components (biomass increment, litter-fall and fine-root turnovers) for NPP estimation is similar to Thomas et al. [47]. The magnitude of NPP estimated in our study differs from other studies. For example, the simulated annual NPP of northeast forests in 2008 by CASA (Carnegie-Ames-Stanford Approach) model ranges from 6.4 gC m^-2^ yr^-1^ to 933.5 gC m^-2^ yr^-1^ with the mean value of 451.6 gC m^-2^ yr^-1^ [22]. The NPP of broad-leaf forests based on field measurements [55] is a little higher than our results. The underestimation of NPP by our method may be due to the exclusion of mortality and understory NPP. But for coniferous, our results are consistent with NPP based on field measurements [55]. Wang [14] showed that mean annual NPP of deciduous needle-leaf forests was 424.7 (gC m^-2^ yr^-1^) in Heilongjiang province. In this study, due to the lack of data, we assume that carbon allocated to standing dead trees is a very small proportion of whole stand NPP and that understory NPP does not affect the normalized NPP-age curves. Zhou et al. [56] found that the understory carbon contents for three old-growth forests on Changbai Mountains accounted for only less than 2% of total carbon storage was relatively stable over time. These results appear to differ from those of Chen et al. [36] who showed that understory NPP exponentially declines with ages.

In addition to biomass model errors, there are several other sources of errors: (1) the average diameter at breast height and the average tree height used in biomass models give a smooth biomass curve under mean environmental conditions. The accuracy in these diameter and height data is affected by uncertainties from yield tables; (2) specie-specific leaf or fine-root turnover rates and the ratio of new fine root carbon to new leaf carbon allocation are found from literature for the same genus elsewhere, and these literature values may not be representative of the species best in our study area; (3) the assumption that LAI does not vary with age after reaching its maximum may not always be true and may have an effect on NPP at old ages; and (4) stand ages in this study are limited by ranges of stand ages in the yield table, which produced errors on biomass and NPP fittings. The total uncertainty in the NPP-age curve in consideration of all these factors is estimated about 25%, and it increases with age. However, since the bias in NPP estimation caused by a factor is mostly uniform across the age spectrum, we expect that the normalized curves (Fig 6(a)) would be most reliable for carbon cycle modeling.

## Conclusions

Based on yield tables from the Heilongjiang Province in northeast China, we derived forest type-dependent NPP-age curves under different SCIs. The following species are considered in our study: *Pinus koraiensis*, *Pinus sylvestrisvar*, *Larix gmelinii*, *Picea koraiensis*, *Populus davidiana*, *Quercus mongolica*, *Betula davuria*, *Tilia amurensis*, and *Betula platyphylla*. We found that NPP increases quickly in young forests, and then decreases at various rates after reaching its maximum at mid ages. Trees in better site conditions are more productive at young ages and their NPP decreases more sharply at old ages. The age at which NPP reaches its maximum value is earlier in better site conditions. The relative variations of NPP with age in poor site conditions are smaller than those in good site conditions. NPP of old forests (> 100 years) maintains about 30–60% of the maximum NPP occurred at mid-ages (15–40 years). The normalized NPP-age relationships are much less sensitive to SCIs, within 10% for most species.

These findings are achieved in China, and confirm the researches previously conducted in USA and Canada. The results produced in this paper will be useful for carbon cycle modelers to decide specific NPP-age curves based on SCIs and also will be helpful for foresters to decide the most suitable curve for use in a particular area.

## Appendix A

Empirical models for the total biomass and the biomass of six components including aboveground, underground, stem, crown, branch, and foliage biomass.

$$lnB_{t}=lna_{1}+b_{1}lnD+c_{1}lnH$$ (10)

$${\text{B}}_{\text{a}}=\frac{1}{1+{\text{r}}_{1}{\text{D}}^{{\text{r}}_{\text{2}}}{\text{H}}^{{\text{r}}_{\text{3}}}}{\text{B}}_{\text{t}}$$ (11)

$${\text{B}}_{\text{r}}=\frac{{\text{r}}_{1}{\text{D}}^{{\text{r}}_{\text{2}}}{\text{H}}^{{\text{r}}_{\text{3}}}}{1+{\text{r}}_{1}{\text{D}}^{{\text{r}}_{\text{2}}}{\text{H}}^{{\text{r}}_{\text{3}}}}{\text{B}}_{\text{t}}$$ (12)

$${\text{B}}_{\text{s}}=\frac{{\text{r}}_{1}{\text{D}}^{{\text{r}}_{\text{2}}}{\text{H}}^{{\text{r}}_{\text{3}}}}{1+{\text{r}}_{1}{\text{D}}^{{\text{r}}_{\text{2}}}{\text{H}}^{{\text{r}}_{\text{3}}}}{\text{B}}_{\text{a}}$$ (13)

$${\text{B}}_{\text{c}}=\frac{1}{1+{\text{r}}_{1}{\text{D}}^{{\text{r}}_{\text{2}}}{\text{H}}^{{\text{r}}_{\text{3}}}}{\text{B}}_{\text{a}}$$ (14)

$${\text{B}}_{\text{b}}=\frac{1}{1+{\text{r}}_{1}{\text{D}}^{{\text{r}}_{\text{2}}}}{\text{B}}_{\text{c}}$$ (15)

$${\text{B}}_{\text{f}}=\frac{{\text{r}}_{1}{\text{D}}^{{\text{r}}_{\text{2}}}}{1+{\text{r}}_{1}{\text{D}}^{{\text{r}}_{\text{2}}}}{\text{B}}_{\text{c}}$$ (16)

## Supporting information

S1 File Yield table was used in this study. (DOCX)
