# Supplementary material for: Influence of site index on the relationship between forest net primary productivity and stand age
Source: PLoS One. 2017 May 11;12(5):e0177084. doi: 10.1371/journal.pone.0177084 (PMC5426654; doi:10.1371/journal.pone.0177084)
Supplement: S1 File — (DOCX) [file pone.0177084.s001.docx]

Yield table of *Betula platyphylla* (SCI=14m)

| age（a） | height（m） | Diameter at breast height（cm） | density（trees/hm^2^） | Basal area（m^2^/hm^2^） | volume（m^3^/hm^2^） | Volume increments（m^3^/hm^2^﹒a） |
| --- | --- | --- | --- | --- | --- | --- |
| 10 | 6.08 | 5.7 | 1231 | 3.11 | 10.5 | 1.05 |
| 15 | 7.84 | 7.2 | 1186 | 4.89 | 20.1 | 1.34 |
| 20 | 9.36 | 8.6 | 1128 | 6.57 | 30.7 | 1.54 |
| 25 | 10.69 | 9.8 | 1068 | 8.13 | 41.8 | 1.67 |
| 30 | 11.90 | 11.0 | 1009 | 9.55 | 52.8 | 1.76 |
| 35 | 12.99 | 12.0 | 954 | 10.84 | 63.5 | 1.81 |
| 40 | 14.00 | 13.0 | 903 | 11.99 | 73.7 | 1.84 |
| 45 | 14.93 | 13.9 | 856 | 13.03 | 83.3 | 1.85 |
| 50 | 15.79 | 14.8 | 813 | 13.97 | 92.4 | 1.85 |
| 55 | 16.59 | 15.6 | 773 | 14.81 | 100.9 | 1.83 |
| 60 | 17.34 | 16.4 | 736 | 15.56 | 108.9 | 1.81 |
| 65 | 18.04 | 17.2 | 702 | 16.25 | 116.3 | 1.79 |
| 70 | 18.70 | 17.9 | 671 | 16.87 | 123.2 | 1.76 |

Yield table of *Betula davuria* (SCI=14m)

| age（a） | height（m） | Diameter at breast height（cm） | density（trees/hm^2^） | Basal area（m^2^/hm^2^） | volume（m^3^/hm^2^） | Volume increments（m^3^/hm^2^﹒a） |
| --- | --- | --- | --- | --- | --- | --- |
| 10 | 5.95 | 7.5 | 1930 | 8.49 | 31.3 | 3.13 |
| 15 | 7.84 | 9.1 | 1562 | 10.23 | 42.8 | 2.85 |
| 20 | 9.44 | 10.5 | 1345 | 11.68 | 52.6 | 2.63 |
| 25 | 10.82 | 11.7 | 1199 | 12.93 | 61.2 | 2.45 |
| 30 | 12.02 | 12.8 | 1092 | 14.05 | 68.8 | 2.29 |
| 35 | 13.07 | 13.8 | 1009 | 15.06 | 75.7 | 2.16 |
| 40 | 14.00 | 14.7 | 943 | 15.98 | 82.0 | 2.05 |
| 45 | 14.82 | 15.5 | 889 | 16.84 | 87.8 | 1.95 |
| 50 | 15.55 | 16.3 | 843 | 17.65 | 93.2 | 1.86 |
| 55 | 16.21 | 17.1 | 804 | 18.40 | 98.3 | 1.79 |
| 60 | 16.79 | 17.8 | 771 | 19.11 | 103.0 | 1.72 |
| 65 | 17.31 | 18.4 | 741 | 19.79 | 107.4 | 1.65 |
| 70 | 17.77 | 19.1 | 715 | 20.43 | 111.6 | 1.59 |

Yield table of *Populus* plantation (SCI=14m)

| age（a） | height（m） | Diameter at breast height（cm） | density（trees/hm^2^） | Basal area（m^2^/hm^2^） | volume（m^3^/hm^2^） | Volume increments（m^3^/hm^2^﹒a） |
| --- | --- | --- | --- | --- | --- | --- |
| 6 | 7.81 | 7.8 | 1500 | 7.2 | 44.2 | 7.37 |
| 8 | 9.11 | 9.3 | 1438 | 9.79 | 61.4 | 7.68 |
| 10 | 10.21 | 10.6 | 1378 | 12.06 | 76.7 | 7.67 |
| 12 | 11.17 | 11.6 | 1321 | 14.07 | 90.4 | 7.54 |
| 14 | 12.01 | 12.6 | 1266 | 15.85 | 102.7 | 7.33 |
| 16 | 12.75 | 13.5 | 1214 | 17.45 | 113.7 | 7.1 |
| 18 | 13.41 | 14.4 | 1163 | 18.87 | 123.5 | 6.86 |
| 20 | 14.00 | 15.2 | 1115 | 20.14 | 132.4 | 6.62 |
| 22 | 14.53 | 15.9 | 1069 | 21.28 | 140.3 | 6.38 |
| 24 | 15.01 | 16.6 | 1024 | 22.3 | 147.4 | 6.14 |
| 26 | 15.44 | 17.3 | 982 | 23.2 | 153.8 | 5.92 |
| 28 | 15.84 | 18.0 | 941 | 24.01 | 159.5 | 5.7 |
| 30 | 16.19 | 18.7 | 902 | 24.73 | 164.5 | 5.48 |

Yield table of *Populus davidiana* (SCI=14m)

| age（a） | height（m） | Diameter at breast height（cm） | density（trees/hm^2^） | Basal area（m^2^/hm^2^） | volume（m^3^/hm^2^） | Volume increments（m^3^/hm^2^﹒a） |
| --- | --- | --- | --- | --- | --- | --- |
| 10 | 7.29 | 8.1 | 1581 | 8.17 | 32.9 | 3.29 |
| 15 | 9.51 | 9.9 | 1506 | 11.53 | 56.9 | 3.79 |
| 20 | 11.31 | 11.4 | 1393 | 14.18 | 79.1 | 3.96 |
| 25 | 12.78 | 12.7 | 1283 | 16.32 | 99.1 | 3.96 |
| 30 | 14.00 | 14.0 | 1185 | 18.12 | 116.9 | 3.90 |
| 35 | 15.02 | 15.1 | 1101 | 19.68 | 132.9 | 3.80 |
| 40 | 15.87 | 16.1 | 1030 | 21.09 | 147.4 | 3.68 |
| 45 | 16.59 | 17.1 | 970 | 22.37 | 160.7 | 3.57 |
| 50 | 17.19 | 18.1 | 918 | 23.57 | 173.0 | 3.46 |
| 55 | 17.70 | 19.0 | 874 | 24.69 | 184.5 | 3.36 |
| 60 | 18.14 | 19.8 | 835 | 25.76 | 195.3 | 3.25 |
| 65 | 18.50 | 20.6 | 800 | 26.78 | 205.4 | 3.16 |
| 70 | 18.81 | 21.4 | 770 | 27.75 | 215.0 | 3.07 |
| 75 | 19.07 | 22.2 | 743 | 28.69 | 224.0 | 2.99 |
| 80 | 19.30 | 22.9 | 718 | 29.59 | 232.6 | 2.91 |

Yield table of *Quercus mongolica* (SCI=14m)

| age（a） | height（m） | Diameter at breast height（cm） | density（trees/hm^2^） | Basal area（m^2^/hm^2^） | volume（m^3^/hm^2^） | Volume increments（m^3^/hm^2^﹒a） |
| --- | --- | --- | --- | --- | --- | --- |
| 10 | 6.27 | 5.7 | 3704 | 9.48 | 36.1 | 3.61 |
| 15 | 7.80 | 7.5 | 2705 | 12.09 | 54.0 | 3.60 |
| 20 | 9.06 | 9.2 | 2171 | 14.33 | 71.1 | 3.55 |
| 25 | 10.14 | 10.6 | 1834 | 16.32 | 87.3 | 3.49 |
| 30 | 11.09 | 12.0 | 1601 | 18.13 | 102.7 | 3.42 |
| 35 | 11.93 | 13.3 | 1430 | 19.79 | 117.3 | 3.35 |
| 40 | 12.69 | 14.5 | 1298 | 21.33 | 131.3 | 3.28 |
| 45 | 13.38 | 15.6 | 1193 | 22.76 | 144.5 | 3.21 |
| 50 | 14.00 | 16.6 | 1108 | 24.11 | 157.2 | 3.14 |
| 55 | 14.57 | 17.7 | 1037 | 25.38 | 169.3 | 3.08 |
| 60 | 15.09 | 18.6 | 976 | 26.58 | 180.9 | 3.01 |
| 65 | 15.57 | 19.5 | 925 | 27.71 | 191.9 | 2.95 |
| 70 | 16.02 | 20.4 | 880 | 28.79 | 202.5 | 2.89 |
| 75 | 16.43 | 21.2 | 841 | 29.82 | 212.6 | 2.83 |
| 80 | 16.81 | 22.1 | 807 | 30.81 | 222.3 | 2.78 |
| 85 | 17.16 | 22.8 | 776 | 31.74 | 231.6 | 2.72 |
| 90 | 17.49 | 23.6 | 749 | 32.64 | 240.5 | 2.67 |
| 95 | 17.79 | 24.3 | 724 | 33.50 | 249.1 | 2.62 |
| 100 | 18.08 | 25.0 | 701 | 34.33 | 257.3 | 2.57 |

Yield table of *Tilia* (SCI=14m)

| age（a） | height（m） | Diameter at breast height（cm） | density（trees/hm^2^） | Basal area（m^2^/hm^2^） | volume（m^3^/hm^2^） | Volume increments（m^3^/hm^2^﹒a） |
| --- | --- | --- | --- | --- | --- | --- |
| 10 | 5.49 | 6.1 | 1763 | 5.11 | 19.0 | 1.90 |
| 15 | 7.29 | 7.8 | 1693 | 8.02 | 35.3 | 2.35 |
| 20 | 8.78 | 9.2 | 1590 | 10.64 | 51.8 | 2.59 |
| 25 | 10.03 | 10.5 | 1482 | 12.94 | 67.2 | 2.69 |
| 30 | 11.09 | 11.7 | 1379 | 14.95 | 81.4 | 2.71 |
| 35 | 11.99 | 12.9 | 1285 | 16.71 | 94.2 | 2.69 |
| 40 | 12.77 | 13.9 | 1201 | 18.26 | 105.7 | 2.64 |
| 45 | 13.43 | 14.9 | 1126 | 19.65 | 116.1 | 2.58 |
| 50 | 14.00 | 15.8 | 1060 | 20.91 | 125.7 | 2.51 |
| 55 | 14.49 | 16.7 | 1002 | 22.07 | 134.4 | 2.44 |
| 60 | 14.91 | 17.6 | 950 | 23.14 | 142.5 | 2.37 |
| 65 | 15.28 | 18.4 | 904 | 24.14 | 150.0 | 2.31 |
| 70 | 15.59 | 19.2 | 862 | 25.08 | 157.0 | 2.24 |
| 75 | 15.86 | 20.0 | 826 | 25.97 | 163.7 | 2.18 |
| 80 | 16.10 | 20.8 | 792 | 26.82 | 169.9 | 2.12 |

Yield table of *Larix* (SCI=14m)

| age（a） | height（m） | Diameter at breast height（cm） | density（trees/hm^2^） | Basal area（m^2^/hm^2^） | volume（m^3^/hm^2^） | Volume increments（m^3^/hm^2^﹒a） |
| --- | --- | --- | --- | --- | --- | --- |
| 8 | 5.64 | 5.1 | 2200 | 4.41 | 14.3 | 1.78 |
| 10 | 6.68 | 6.5 | 2138 | 7.06 | 26.4 | 2.64 |
| 12 | 7.63 | 7.6 | 2077 | 9.53 | 39.9 | 3.33 |
| 14 | 8.53 | 8.6 | 2019 | 11.86 | 54.4 | 3.89 |
| 16 | 9.36 | 9.5 | 1962 | 14.04 | 69.6 | 4.35 |
| 18 | 10.15 | 10.4 | 1906 | 16.09 | 85.1 | 4.73 |
| 20 | 10.88 | 11.1 | 1852 | 18.02 | 100.7 | 5.03 |
| 22 | 11.58 | 11.8 | 1800 | 19.82 | 116.2 | 5.28 |
| 24 | 12.24 | 12.5 | 1749 | 21.50 | 131.6 | 5.48 |
| 26 | 12.86 | 13.1 | 1700 | 23.07 | 146.6 | 5.64 |
| 28 | 13.44 | 13.8 | 1652 | 24.53 | 161.2 | 5.76 |
| 30 | 14.00 | 14.3 | 1605 | 25.88 | 175.3 | 5.84 |
| 32 | 14.53 | 14.9 | 1560 | 27.13 | 188.8 | 5.9 |
| 34 | 15.03 | 15.4 | 1515 | 28.29 | 201.8 | 5.94 |
| 36 | 15.50 | 15.9 | 1473 | 29.35 | 214.1 | 5.95 |
| 38 | 15.95 | 16.4 | 1431 | 30.33 | 225.9 | 5.94 |
| 40 | 16.38 | 16.9 | 1391 | 31.22 | 236.9 | 5.92 |

Yield table of *Pinus koraiensis* (SCI=12m)

| age（a） | height（m） | Diameter at breast height（cm） | density（trees/hm^2^） | Basal area（m^2^/hm^2^） | volume（m^3^/hm^2^） | Volume increments（m^3^/hm^2^﹒a） |
| --- | --- | --- | --- | --- | --- | --- |
| 10 | 2.87 | 4.9 | 2200 | 4.13 | 11.9 | 1.19 |
| 12 | 4.10 | 7.0 | 2167 | 8.32 | 27.0 | 2.25 |
| 14 | 5.30 | 8.6 | 2135 | 12.39 | 43.1 | 3.08 |
| 16 | 6.42 | 9.9 | 2103 | 16.25 | 59.1 | 3.70 |
| 18 | 7.45 | 11.0 | 2071 | 19.78 | 74.2 | 4.12 |
| 20 | 8.39 | 11.9 | 2040 | 22.86 | 87.6 | 4.38 |
| 22 | 9.25 | 12.7 | 2010 | 25.45 | 99.1 | 4.50 |
| 24 | 10.03 | 13.3 | 1980 | 27.57 | 108.7 | 4.53 |
| 26 | 10.75 | 13.8 | 1950 | 29.27 | 116.5 | 4.48 |
| 28 | 11.40 | 14.3 | 1921 | 30.64 | 123.0 | 4.39 |
| 30 | 12.00 | 14.6 | 1893 | 31.75 | 128.3 | 4.28 |
| 32 | 12.55 | 14.9 | 1864 | 32.65 | 132.7 | 4.15 |
| 34 | 13.05 | 15.2 | 1836 | 33.39 | 136.3 | 4.01 |
| 36 | 13.52 | 15.5 | 1809 | 34.01 | 139.4 | 3.87 |
| 38 | 13.95 | 15.7 | 1782 | 34.52 | 142.0 | 3.74 |
| 40 | 14.35 | 15.9 | 1755 | 34.95 | 144.2 | 3.61 |
| 42 | 14.72 | 16.1 | 1729 | 35.32 | 146.1 | 3.48 |
| 44 | 15.07 | 16.3 | 1703 | 35.64 | 147.8 | 3.36 |
| 46 | 15.39 | 16.5 | 1678 | 35.91 | 149.2 | 3.24 |
| 48 | 15.69 | 16.7 | 1653 | 36.14 | 150.5 | 3.14 |
| 50 | 15.98 | 16.9 | 1628 | 36.35 | 151.6 | 3.03 |

Yield table of *Pinus sylvestris* (SCI=14m)

| age（a） | height（m） | Diameter at breast height（cm） | density（trees/hm^2^） | Basal area（m^2^/hm^2^） | volume（m^3^/hm^2^） | Volume increments（m^3^/hm^2^﹒a） |
| --- | --- | --- | --- | --- | --- | --- |
| 10 | 4.37 | 6.9 | 2200 | 8.14 | 24.7 | 2.47 |
| 12 | 5.85 | 8.4 | 2127 | 11.65 | 39.4 | 3.28 |
| 14 | 7.20 | 9.6 | 2057 | 14.78 | 53.4 | 3.81 |
| 16 | 8.41 | 10.6 | 1988 | 17.60 | 66.4 | 4.15 |
| 18 | 9.50 | 11.6 | 1923 | 20.16 | 78.4 | 4.35 |
| 20 | 10.47 | 12.4 | 1859 | 22.49 | 89.4 | 4.47 |
| 22 | 11.33 | 13.2 | 1797 | 24.63 | 99.6 | 4.53 |
| 24 | 12.10 | 14.0 | 1738 | 26.59 | 109.0 | 4.54 |
| 26 | 12.80 | 14.7 | 1680 | 28.38 | 117.6 | 4.52 |
| 28 | 13.43 | 15.3 | 1624 | 30.02 | 125.5 | 4.48 |
| 30 | 14.00 | 16.0 | 1571 | 31.51 | 132.8 | 4.43 |
| 32 | 14.52 | 16.6 | 1519 | 32.88 | 139.4 | 4.36 |
| 34 | 14.99 | 17.2 | 1468 | 34.13 | 145.5 | 4.28 |
| 36 | 15.43 | 17.8 | 1420 | 35.27 | 151.1 | 4.20 |
| 38 | 15.82 | 18.4 | 1373 | 36.30 | 156.1 | 4.11 |
| 40 | 16.19 | 18.9 | 1327 | 37.23 | 160.7 | 4.02 |
| 42 | 16.53 | 19.4 | 1283 | 38.07 | 164.9 | 3.93 |
| 44 | 16.85 | 20.0 | 1241 | 38.83 | 168.6 | 3.83 |
| 46 | 17.14 | 20.5 | 1199 | 39.50 | 172.0 | 3.74 |
| 48 | 17.41 | 21.0 | 1160 | 40.09 | 175.0 | 3.65 |
| 50 | 17.67 | 21.5 | 1121 | 40.62 | 177.7 | 3.55 |
